# Optical–Electrical Coordinately Modulated Memristor Based on 2D Ferroelectric RP Perovskite for Artificial Vision Applications

**DOI:** 10.1002/advs.202403150

**Published:** 2024-07-01

**Authors:** Hong Wang, Jialiang Yang, Zheng Yang, Gongjie Liu, Yusong Tang, Yiduo Shao, Xiaobing Yan

**Affiliations:** ^1^ Key Laboratory of Brain‐Like Neuromorphic Devices and Systems of Hebei Province Hebei Key Laboratory of Photo‐Electricity Information and Materials Hebei University Baoding 071002 China; ^2^ Department of Materials Science and Engineering National University of Singapore Singapore 117576 Singapore

**Keywords:** 2D ferroelectricity, 2D Ruddlesden‐Popper perovskite, biological synapse, memristor, visual perception

## Abstract

Traditional artificial vision systems built using separate sensing, computing, and storage units have problems with high power consumption and latency caused by frequent data transmission between functional units. An effective approach is to transfer some memory and computing tasks to the sensor, enabling the simultaneous perception‐storage‐processing of light signals. Here, an optical–electrical coordinately modulated memristor is proposed, which controls the conductivity by means of polarization of the 2D ferroelectric Ruddlesden–Popper perovskite film at room temperature. The residual polarization shows no significant decay after 10^9^‐cycle polarization reversals, indicating that the device has high durability. By adjusting the pulse parameters, the device can simulate the bio‐synaptic long/short‐term plasticity, which enables the control of conductivity with a high linearity of ≈0.997. Based on the device, a two‐layer feedforward neural network is built to recognize handwritten digits, and the recognition accuracy is as high as 97.150%. Meanwhile, building optical–electrical reserve pool system can improve 14.550% for face recognition accuracy, further demonstrating its potential for the field of neural morphological visual systems, with high density and low energy loss.

## Graphical abstract

2D (BA)_2_(MA)_3_Pb_4_Cl_13_ perovskite is ferroelectric at room temperature, and its residual polarization reversal can reach 10^9^ cycles. Based on 2D perovskite memristors, a 2‐layer feedforward electrical neural network, and a photoelectric reserve pool system are established to recognize the image recognition. The accuracy is separately 97.15% and 90.91%, further demonstrating its potential in the field of neuromorphic visual systems.

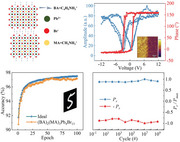

## 1 Introduction

Approximately 80% of the information humans receive from the external world is obtained through visual perception, usually by photoreceptor cells in the retina detecting light signals. The light signals are subsequently converted into electrical signals, which are preprocessed to filter out unnecessary visual data.^[^ ^1^, ^2^ ^]^ Effective light information is transmitted via the optic nerve to the visual cortex for advanced processing. Inspired by this process, optoelectronic devices have been developed to simulate the work process of the retina system, but traditional silicon‐based visual devices require long‐distance data communication from the sensor to the processing and storage unit, leading to problems such as high energy consumption, long response time, and communication bandwidth pressure.^[^ ^3^, ^4^ ^]^ As a result, optoelectronic resistive memory devices with sense and store integrated have been proposed to improve the challenges of data transmission and processing time and energy consumption between sensors, processors, and storage units, enabling fast and efficient machine vision processing. Optoelectronic resistive memories not only make up for the lack of light perception function of traditional storage memory, but also have advantages such as simple structure, fast speed, strong plasticity, low power consumption, and easy integration.^[^ ^5^ ^]^ Therefore, for future artificial vision systems, optoelectronic memristor can be integrated on chips, not only having computing functions but also having temporary memory and real‐time processing ability of visual information. Therefore, the development of a sensing‐storage‐computing artificial vision system is crucial for high‐speed and energy‐efficient information processing.

Currently, most of the photoelectric memristors are filament type, and the formation and breakage of conductive filaments (CF) are considered to be the main cause of their resistance variation. Some devices have temperature‐dependent phenomena due to the Joule heat that may cause the breakage of CF.^[^ ^6^, ^7^, ^8^ ^]^ Ferroelectric materials have controlled spontaneous polarization under applied electric field,^[^ ^9^, ^10^ ^]^ The ferroelectric materials have shown great potential for optoelectronic and information storage applications and their use as light‐absorbing layers in ferroelectric photovoltaic devices.^[^ ^11^, ^12^ ^]^ In recent years, perovskite‐like organic‐inorganic compound have been extensively investigated in photovoltaic devices due to their remarkable optical, ferroelectric and piezoelectric properties.^[^ ^13^, ^14^, ^15^, ^16^ ^]^ Compared to 3D perovskites, 2D Ruddlesden‐Popper (RP) perovskites are endowed with ferroelectric functions due to their structural diversity and tunability.^[^ ^17^ ^]^ For example, (BA)_2_(MA)_2_Pb_3_Cl_10_ (BA = C_4_H_9_NH_3_^+^), the combination of ferroelectric and excellent optoelectronic properties yields optoelectronic ferroelectrics with switchable spontaneous polarization.^[^ ^18^ ^]^ Despite the excellent performance of perovskites in different aspects, the stability and durability of their ferroelectric polarization are still crucial issues.^[^ ^19^ ^]^ Usually the polarization fatigue of a device is defined as the degradation or suppression of the device polarization after a large number of electrical loading cycles, which determines the lifetime and stability of the device. *Kang* et al. showed that the polarization value of Bi_3_TiTaO_9_ perovskite films degraded to approximately 60% of the initial value after 3 × 10^7^ switching cycles.^[^ ^20^ ^]^ Chae et al. reported metal‐doped PZT films with device polarization values decreasing slowly around 10^6^ continually to 10^9^ fatigue cycles.^[^ ^21^ ^]^ As previously reported by *Wang* et al.,^[^ ^22^ ^]^ demonstrated a 2D RP perovskite thin‐film device, a structured device with excellent optoelectronic properties but with a large and asymmetric coercivity field voltage. Therefore, in this paper, we explore the optoelectronic properties of the double‐ended memristor device by using a conventional three‐layer structure to enhance its ferroelectric polarization durability performance.

In this work, 2D ferroelectric perovskite (BA)_2_(MA)_3_Pb_4_Cl_13_ (BA = C_4_H_9_NH_3_^+^, MA = CH_3_NH_3_^+^) films were prepared using spin–coating vacuum heating method and designed Pd/(BA)_2_(MA)_3_Pb_4_Cl_13_/SiO_2_/Si structure of the memristor. First, the two‐dimensional perovskite was confirmed to be ferroelectric using piezoelectric force microscopy (PFM) and a ferroelectric tester, and its anti‐fatigue properties were tested. The device was verified to have typical memristive resistance switching behavior, and the device conductance was regulated by tuning the pulse parameters. It can simulate long‐term and short‐term synaptic plasticity, including post‐tetanic potentiation (PTP), paired‐pulse facilitation/depression (PPF/PPD), long‐term potentiation/depression (LTP/LTD), and learned forgetting properties. Handwritten digit recognition was subsequently performed with an accuracy of 97.15%. The device has different degrees of light response in the visible band and good stability performance at the same wavelength. At the same time, the influence of visible light irradiation on the device response current during the application of electrical pulses was also explored, demonstrating the potential for visual perception of perovskite devices.

## 2 Results and Discussion

**Figure 1:**
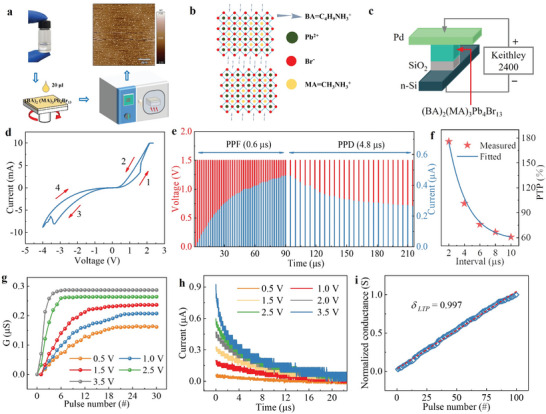
Electrical characteristics of 2D (BA)_2_(MA)_3_Pb_4_Br_13_ memristors. a) Schematic diagram of 2D RP perovskite film preparation process. The illustration is an AFM image of 2D (BA)2(MA)3Pb4Br13 films b) 2D (BA)2(MA)3Pb4Br13 perovskite crystal structure. c) Schematic diagram of the Pd/(BA)2(MA)3Pb4Cl13/SiO2/Si structure. d) I–V characteristic curve of 2D (BA)2(MA)3Pb4Br13 memristors. e) PPF (1.5 V, 0.6 µs) and PPD (1.5 V, 4.8 µs). f) PTP test results and fitted curves. g,h) Conductance changes and EPSC of the devices with different amplitude pulses (0.5, 1.0, 1.5, 2.5, 3.5; 0.5, 1.0, 1.5, 2.0, 2.5, 3.5 V). i) LTP of the devices.

The Pd/(BA)_2_(MA)_3_Pb_4_Cl_13_/SiO_2_/Si memristor was prepared using spin–coating and magnetron sputtering techniques. The SiO_2_ bandgap is ≈8.9 eV, and our intention is to build a certain barrier height with 2D (BA)_2_(MA)_3_Pb_4_Cl_13_ perovskite to reduce devices power consumption. The SiO_2_ preparation process in the device is consistent with our previously reported work,^[^ ^23^ ^]^ and the thickness of SiO_2_ is ≈3.4 nm. We provide a schematic diagram of the preparation process of 2D RP perovskite films using spin coating method, as shown in **Figure** **1**a. The detailed preparation process is described in the experimental section. To demonstrate the flatness of the film, the atomic force microscope (AFM) image is obtained and shown in the upper right corner of Figure 1a. Meanwhile, the (BA)_2_(MA)_3_Pb_4_Cl_13_ atomic structure is shown in Figure 1b, and its corresponding device structure is also shown in Figure 1c. The Pd (≈14 nm) preparation process and its transparency have been publicly reported in our previous work,^[^ ^24^ ^]^ and will not be elaborated here. The I–V characteristic curve of the device was tested, exhibiting typical resistive switching behavior as shown in Figure 1d. Applying a cyclic voltage of 0 V→2 V→0 V→−4 V→0 V to the device, the device exhibits a positive transition from HRS to LRS as the positive voltage gradually increases; when a negative voltage scan is performed, the device achieves a transition from LRS to HRS as the voltage slowly increases. Figure S1a (Supporting Information) illustrates the logarithmic form of I–V, showing the classical switching performance of the memristor. Figure S1b (Supporting Information) shows the high and low resistance retention of the device over 10^4^ s, demonstrating the stability of the perovskite device. Figure S2 (Supporting Information) shows the unidirectional sweeping of the device in the positive and negative directions, respectively. The device current decreases as the number of turns increases, and the device resistance gradually increases. This result demonstrates that the device has a good ability to regulate the bi‐directional conductance.^[^ ^25^ ^]^

The I–V test results show that the device resistance can be adjusted in response to voltage, a property that provides the prerequisite for the device to achieve an electrical pulse response. The top electrode can be functionally considered as the presynaptic membrane, the bottom electrode corresponds to the postsynaptic membrane, and the (BA)_2_(MA)_3_Pb_4_Cl_13_ functional layer serves as the synaptic gap. The device PPF to PPD transition is investigated below by designing a series of pulses with different parameters to measure the response of the device.^[^ ^25^, ^26^ ^]^ The pulse amplitude is fixed at 1.5 V, pulse width is 1.2 µs, pulse interval is fixed at 0.6 µs, and the number of pulses is 50. After that, 25 pulses are then set (pulse amplitude and pulse width parameters remain unchanged, and pulse interval is set at 4.8 µs), and the test results are shown in Figure 1e shows that in the first 50 pulses, the current value of the device is in a continuous process of becoming larger as the number of pulses increases. When the pulse interval changes to 4.8 µs, the current value of the device is in a continuous process of decreasing, leading to PPD, which is similar to the brain's memory‐to‐forget process.^[^ ^27^ ^]^ Ten pulses were set (pulse width was fixed at 1 µs and pulse amplitude was fixed at 2 V). The PTP was calculated as:

(1) $$\mathrm{PTP}=\frac{G_{10}-G_{1}}{G_{1}}\times 100\%$$

*G_10_* indicates the conductance value after the tenth pulse stimulation. The results are shown in Figure 1f shows that the relaxation time constants *τ_1_* and *τ_2_* of the fitted results are 1.7 and 2.0 µs, respectively. All of the above studies demonstrate that the device has good properties of simulating biological synapses.

Applying a continuous pulse train to explore the response of the device is important for achieving synaptic plasticity.^[^ ^28^ ^]^ First, different forms of programmed pulses (varying the amplitude, duration, and interval of the pulses) were applied to the device, as shown in Figure 1g,h. It can be seen that the device conductance value increases with increasing pulse amplitude and the excitatory postsynaptic current (EPSC) increases with increasing amplitude. As shown in Figure S3a,c (Supporting Information), it can be seen that the device conductance increases with increasing pulse duration, and its EPSC likewise increases with increasing pulse duration. Conversely, the device conductance and EPSC decrease with increasing pulse interval, as shown in Figure S3b,d (Supporting Information). The study shows that the EPSC of the device not only becomes more current but also decays more slowly as the pulse amplitude increases. The conductance bidirectional variation of 2D (BA)_2_(MA)_3_Pb_4_Br_13_ devices in pulse mode is investigated to simulate the synaptic LTP/LTD function. 100 continuous pulses (3 V, 200 ns) with the pulse interval from 200 to 596 ns, were applied to the memristor. As the number of pulses increases, the device conductivity increases linearly as shown in Figure 1i, which is consistent with the biological LTP behavior. We perform linear fitting on it, and its linearity is ≈0.997. The consecutive pulse (–3 V, 1300 ns) with the pulse width from 3300 to 330 ns is measured, corresponding LTD behavior which is shown in Figure S4a (Supporting Information). The conductance of the device is linearly related to the increase of the number of pulses. and its linearity is ≈0.770. The above results indicate that the 2D RP perovskite device has bidirectional regulation properties. The reason why the device can simulate the biological LTP/LTD behavior may be due to the non‐volatile polarization switching and stable mixed domain state^[^ ^29^, ^30^ ^]^ in the 2D ferroelectric (BA)_2_(MA)_3_Pb_4_Br_13_ layer, as shown in Figure S4b,c (Supporting Information). Moreover, the device works differently from traditional memristors, relying on defects and/or charge trapping/detrapping,^[^ ^31^, ^32^, ^33^ ^]^ mainly due to the barrier height of its SiO_2_ configuration caused by polarization flipping. A more advanced synaptic function in synaptic plasticity is the “learning‐forgetting‐relearning” process. This process is usually described by the Ebbinghaus forgetting curve, in which memory is strengthened by repeated learning.^[^ ^34^, ^35^ ^]^ Our artificial synapse based on (BA)_2_(MA)_3_Pb_4_Cl_13_ mimics this behavior by Figure S5 (Supporting Information). It can be seen that the number of stimuli required for the device to recover memory after applying 5 stimulus voltages in the relearning part of the figure is much less than for initial learning.

**Figure 2:**
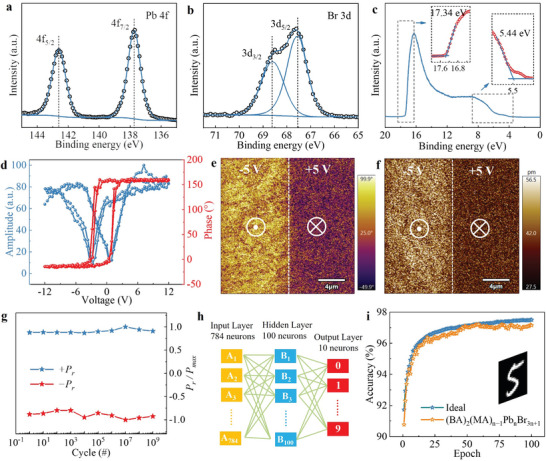
2D ferroelectric (BA)2(MA)3Pb4Cl13 characterization and image recognition application. a,b) XPS local spectrums of the Pb (4f) and Br (3d) elements. c) UPS spectrum of 2D (BA)2(MA)3Pb4Cl13. The illustration shows its local magnification. d) PFM phase and amplitude hysteresis loops. e,f) PFM phase and amplitude images. g) Ferroelectric polarization fatigue characteristics. Pr is the residual polarization value; Pmax is the maximum residual polarization value. h,i) The principle diagram of digital recognition and the recognition accuracy after 100 training sessions, including both ideal and actual situations.

**Figure** **2**a–b are XPS analyses of (BA)_2_(MA)_3_Pb_4_Br_13_ films. Figure 2a shows the core spectrum of Pb 4f, the peaks at 137.7 and 142.5 eV represent Pb 4f_7/2_ and Pb 4f_5/2_ respectively. Figure 2b shows the core spectrum of Br 3d, the peaks at 68.6 and 67.5 eV represent Br 3d_3/2_ and Br 3d_5/2_ respectively. The peaks can be attributed to the Pb‐Br bond, which are consistent with the previously reported data.^[^ ^22^ ^]^ The full XPS spectrum of the perovskite devices is shown in Figure S6 (Supporting Information), and the scans reveal the presence of N, C, Pb, and Br in the prepared perovskite films. The results from the ultraviolet photoelectron spectroscopy (UPS) are shown in Figure 2c, the position of the valence band edge and the Fermi energy level at the top of the film are known, and the Fermi energy level and the valence band edge are located at −4.66 and −6.36 eV, respectively. The ferroelectricity of the structure was then investigated by using PFM to test the thin film, where a square area of 20 µm × 20 µm was written and drawn on the film. A rectangle with a length of 20 µm and a width of 10 µm was drawn on the left and right side of the square area as shown in Figure S7a (Supporting Information), +5 V voltage is applied to the left area, and −5 V voltage is applied to the right area, according to Figure 2e, it can be observed that the PFM domains of (BA)_2_(MA)_3_Pb_4_Br_13_ thin film are obvious, the left region shows yellow color with upward polarization direction, and the right region shows purple color with downward polarization direction. This indicates that applying different bias voltages can flip the polarization direction of ferroelectric domains, thereby achieving directional polarization of ferroelectric domains and the phase flip angle is nearly 150°, which shows the good ferroelectric polarization characteristics of perovskite ferroelectric films. Figure S8c (Supporting Information) is the amplitude change corresponding to the applied voltage. Figure S7b–d (Supporting Information) shows the phase flip images after applying 3 V, 4 V, and 6 V voltages respectively to write the domain operation. From the figures, it can be seen that the phase flip angle is about 123° at 3 V, 135° at 4 V, and 150° at 6 V, which is the same as the flip angle at 5 V. The image of the corresponding amplitude change at the above operating voltages is shown in Figures 2f and S8a–c (Supporting Information). The phase hysteresis curve and the amplitude hysteresis curve shown in Figure 2d can observe the switching reversal of the polarization, and the coercivity voltages are 1.0 and −2.8 V, respectively, as shown by the curves in the figure, showing good ferroelectric reversal characteristics. The memory usually requires multiple read and write operations, so the study of the fatigue characteristics of ferroelectric materials is particularly important.^[^ ^36^ ^]^ Subsequently, the stability performance of the sample was tested and measured by a ferroelectric tester. Figure 2g, showing the relationship between the residual polarization strength and the number of reversals: after 10^9^ polarization reversals, the residual polarization strength of the perovskite device did not decay significantly, reflecting the excellent fatigue resistance of the device. The good ferroelectricity and excellent anti‐fatigue properties lead to the stable simulated biosynaptic function of this perovskite device.^[^ ^37^ ^]^

A feedforward neural network was constructed using devices based on (BA)2(MA)3Pb4Cl13 and tested using a modified national institute of standards and technology (MNIST) dataset^[^ ^38^ ^]^ as shown in Figure 2h shows, where the input, hidden and output layers contain 784 neurons (corresponding to 28 × 28 input pixels), 100 and 10 (corresponding to 10 output categories), respectively. The values obtained from the device LTP test are replaced with the weight matrix in the algorithm, and then the digital recognition process is simulated. Where the linearity of the fitted LTP curve of is 0.997, which is an important factor affecting the accuracy of the digital recognition.^[^ ^39^ ^]^ The accuracy of the test set after 100 training sessions is shown in Figure 2i shows that the accuracy is as high as 97.15% and the perovskite device has high recognition accuracy. The comparison of handwritten digit recognition accuracy for different memristor structures is shown in Table S1 (Supporting Information).

**Figure 3:**
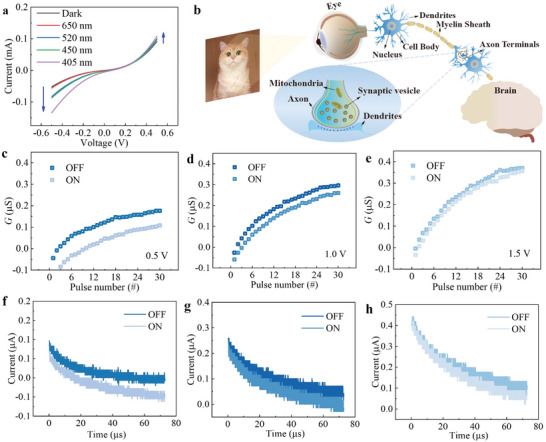
Optical characteristics of 2D (BA)2(MA)3Pb4Br13 devices. a) I–V characteristic curves of devices under the light with different wavelengths (650/520/450/405 nm). b) Schematic diagram of human visual neural network. c–e) Conductance changes of the device under the action of 30 consecutive electrical pulse trains of three amplitudes (0.5, 1.0, 1.5 V) under dark and 650 nm light conditions. f–h) EPSC changes of the device under the action of 30 consecutive electrical pulse trains of three amplitudes (0.5, 1.0, 1.5 V) under dark and 650 nm light conditions.

By applying different wavelengths of visible light (405, 520, 650 nm) on the device respectively, the I‐V characteristic curves of the perovskite films were measured, and it was found that the I‐V currents increased with the decrease of the light wavelength, and the negative direction was more obvious, as shown in **Figure** **3**a. Figure S9 (Supporting Information) demonstrates the I–V variation of the device under different power (27.7 , 138.9, 277.8 mW cm^−2^) of 650 nm wavelength light, from which it can be seen that the device I–V current becomes larger as the power increases. It shows that the device is responsive to visible light and has the potential to simulate artificial vision.^[^ ^40^ ^]^ Figure 3b shows a schematic of neuromorphic vision, the retina on the eye first captures image information and converts it into electrical signals for low‐level preprocessing on the retina. These signals are then transmitted through the optic nerve to the visual cortex, which performs high‐level processing and memory of the acquired image information.^[^ ^41^, ^42^ ^]^ According to Figure 3a results, the wavelength light with the smallest current response, which is 650 nm, and 30 pulses (pulse width and pulse interval were both 2 µs) were set to observe the conductance changes of the device, as shown in Figure 3c–e for the comparison of the device conductance values with and without light under amplitude 0.5  –1.5 V, and it can be seen that the device conductance values still have large changes before and after the addition of light under 650 nm light. Figure 3f–h shows the EPSC variations of Figure 3c–e corresponding to the above 30 pulses. The optical response rate of the device at different wavelengths was tested by Figure S10 (Supporting Information). It can be seen that the difference in the optical response speed at different wavelengths is small. PPF is a form of short‐term synaptic plasticity at biological synapses, in which two excitatory presynaptic pulses are applied consecutively and the second pulse produces a larger excitatory postsynaptic current than the first pulse.^[^ ^28^ ^]^ The result is shown in Figure S11 (Supporting Information). The PPF characteristics of the device were tested in the dark and under light at 405 nm wavelength, demonstrating that the device has suboptical synaptic properties.

**Figure 4:**
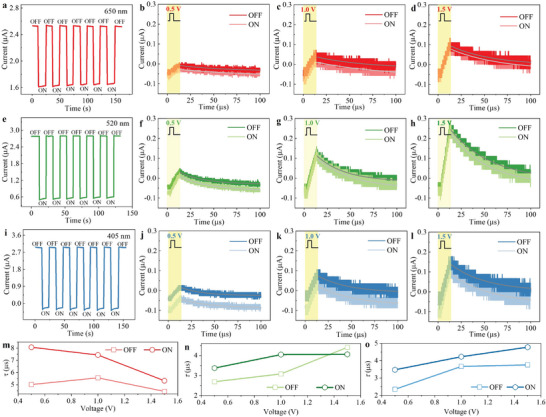
Optical/optical–electrical regulation for 2D (BA)2(MA)3Pb4Br13 memristors. a,e,i) Current responses of the (BA)2(MA)3Pb4Br13 device under the 650/520/405 nm light illumination. b–d, f–h, j–l) Current responses under different single electrical pulse (0.5, 1.0, 1.5 V) under the ON/OFF lights with the 650/520/405 nm wavelength. m,n,o) A plot of relaxation time versus electrical pulse amplitude at the ON or OFF light.

Continue to explore the photovoltaic response of (BA)_2_(MA)_3_Pb_4_Cl_13_ thin film devices under other wavelengths of visible light illumination. First, the devices were applied with a duration of 10 s of 650, 520 and 405 nm light respectively, the duration of no light was also 10 s, and repeated 6 times to observe the current response of the devices as shown in **Figure** **4**a,e,i. It can be seen that the perovskite device has stable photoresponse characteristics at visible wavelengths. Subsequently, an electrical pulse with a pulse width of 10 µs was applied to the device to compare the current response of the device in the presence and absence of light. The positive/negative bidirectional photoresponse current may be caused by the coupling of ferroelectric polarization and photoexcitation.^[^ ^43^ ^–^ ^45^ ^]^ Figure 4b–d shows the current response of the pulses and their EPSCs for the device in dark mode and in the presence of light at 650 nm after applying pulses of amplitude 0.5, 1.0, and 1.5 V to the device, respectively. Figure 4f–h shows the pulse current response and EPSC of the device in dark mode and at 520 nm after applying pulses of 0.5, 1.0, and 1.5 V, respectively. Figure 4j–l shows the pulse current response and EPSC of the device in the dark mode and at 405 nm after applying pulses of 0.5, 1.0, and 1.5 V, respectively. In order to further investigate the simulated short‐term synaptic plasticity under the action of light signals in 2D (BA)_2_(MA)_3_Pb_4_Br_13_ devices, we fitted the current attenuation curves (Figure 4b–d,f–h,j–l) after different parameter signals were applied. The fitting formula is *M(t)* ***=*** *M_i_* ***+*** *(M_m_* **−** *M_i_)exp(* **−** *t/τ)*.^[^ ^46^, ^47^ ^]^ Here, *M_i_* and *M_m_* are separately the initial and steady state of memory, and *τ* is the time constant of the relaxation process. If the *τ* value is larger, indicating a longer decay time of the device current, that the forgetting will be slower. In order to better demonstrate the *τ* value change under different conditions, a plot of *τ* versus electrical pulse amplitude under the ON or OFF light is counted, as shown in Figure 4m–o. The results show that the device forgets slower under a 650 nm light, under the same electrical signal, while forgetting is faster under a 405 nm blue light. The current response of the device after applying electrical pulses of 2.0, 2.5, 3.0, 3.5, and 4.0 V with or without the above visible wavelength light is shown in Figure S12–14 (Supporting Information). From the above test results, it can be seen that the smaller the electrical pulse amplitude of the perovskite device, the larger the change in the device current response after illumination. The smaller the light wavelength, the greater the change in device current response after illumination. These characteristics indicate that the device has research potential in artificial vision. As Figure S15a–d (Supporting Information) shown ln (I) ∼ V_1/4_ all achieve a good linear fit, consistent with the hot electron emission process.^[^ ^48^ ^]^ Figure S16a (Supporting Information) shows the work function, band gap parameters, and potential barrier diagrams for four materials (i.e., Pd, (BA)_2_(MA)_3_Pb_4_Cl_13_, SiO_2_, Si). We thought that the optically‐induced conductivity change might be related to the ferroelectric polarization. To further support that viewpoint, we conducted tests on 2D RP (BA)_2_(MA)_3_Pb_4_Br_13_ perovskite under dark and light conditions using a ferroelectric tester, and obtained typical polarization‐electric‐field (P‐E) loops as shown in the Figure S17 (Supporting Information), in the –4.0–3.7 V range of an electric field at 0.4 MHz probe frequencies. The results show that under the same range of electric field, the ferroelectric polarization intensity of 2D RP (BA)_2_(MA)_3_Pb_4_Br_13_ is greater with light than without light. The maximum forward polarization intensity and residual polarization differ by about 2.92 and 1.62 µC cm^−2^, respectively. The negative direction differs by 2.74 and 0.64 µC cm^−2^. Our phenomenon is consistent with the reported work.^[^ ^49^, ^50^ ^]^ For detailed resistance switching mechanisms, please refer to the supplementary information Figure S16c–d (Supporting Information) and corresponding text.

**Figure 5:**
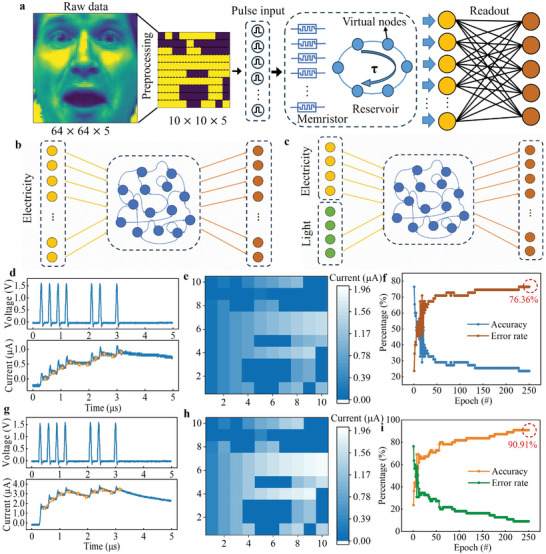
Image recognition system based on 2D (BA)2(MA)3Pb4Cl13 memristor reservoir computational networks. a) Schematic of a neural network reservoir computation for image recognition. b,c) Schematic diagrams of the structure of the computational network for single‐input signals (electrical signals) and mixed‐input signals (optical and electrical signals) in reservoir pools. d,g) The pulse waveforms and response current waveforms are applied corresponding to a single input signal and a mixed input signal. The orange curve shows one of the extracted results of the characteristic current characterization of the reservoir pools (1111001101), which is the sensing and processing of the 10‐bit pulse train pairs. e,h) The face image is converted into a pulse sequence and input into the perovskite memristor‐based reserve pool system to read out the reserve pool status. f,i) Recognition accuracy and error rates of reservoir computing networks under electrical/photoelectric signals for scaled images.

Synapses are a key element in neural networks, and memristor characteristics have laid the foundation for the simulation of neural synapses, which has led to renewed interest in artificial neural networks. It has been shown that memristors are used in neural networks for signal processing, and the key difficulty is to make them have a high recognition rate, which requires high performance of the required devices. The 2D (BA)_2_(MA)_3_Pb_4_Cl_13_‐based device demonstrated excellent switching characteristics in the above tests, offering the possibility of application to reservoir computing (RC) neural networks.^[^ ^51^, ^52^, ^53^, ^54^ ^]^ **Figure** **5** shows an image recognition system using a reservoir computing neural network. Figure 5a shows the process of image recognition with a reservoir computing neural network, in which a photo is first scaled so that the original photo sample is converted into 10 × 10 pixels, and then each row of pixels is converted into a sequence of impulses (the purple area is a logical “0” and the yellow area is a logical “1”), which is used as an input to the reservoir cell. After this process, the pulse sequence signals are converted into analog current signals, and finally, the resulting signals are used for neural network recognition. We perform multiple scaling processes on five people's photos to get multiple 10 × 10 pixel samples, which serve as training samples. The sample images are shown in the support information Figure S18 (Supporting Information). Figure 5b shows a schematic diagram of a conventional single‐input reservoir computing (RC) neural network. For the RC algorithm, multiple electrical signals are transformed into input signals and form a reservoir state, which is computed to realize image recognition. It is worth noting that if a reservoir cell is able to input both optical and electrical signals in order to realize the optoelectronic mixed‐input reservoir computation, as shown in Figure 5c, the output response of the reservoir cell increases, thus the recognition accuracy. Figure 5d,g then shows the results of the output current of the reservoir cell when an electrical signal and an optoelectronic hybrid signal are applied to the device, respectively. Based on the good short‐term synaptic plasticity of the device, both of them can exhibit neural synaptic behaviors. Therefore, the +1.5 V pulse waveforms applied in Figure 5d,g are one of the many waveforms applied. In these waveforms, the display shows a clear current response, and the device exhibits significantly higher current response values when an opto‐electronic hybrid input signal is applied compared to a single signal input. The single input signal has a final output current of 1 µA, while the opto‐electronic hybrid input signal reaches a final output current of 3 µA, which is almost three times that of the electrical signal (blue curve). The reserve cell characteristic current is extracted at 90 ns after each pulse, resulting in a characterization result of 1111001101 (orange curve). Figure 5e,h separately show the face image which is converted into a pulse sequence and input into the perovskite memristor‐based reserve pool system to read out the reserve pool status. After applying the electrical and optoelectronic hybrid signals as input signals to the reserve pool computational neural network, respectively, the results of image recognition are shown in Figure 5f,i. After 250 rounds of learning and training, the recognition rate of the storage pool computational neural network with a single input signal for image recognition can reach 76.36% (Khaki curve in Figure 5f). When an optical signal is added to the input signal, the recognition rate increases significantly and can reach 90.91% (yellow curve in Figure 5i). It can be seen that the application of 2D (BA)_2_(MA)_3_Pb_4_Cl_13_‐based devices in optoelectronic input reservoir neuromorphic computation has good development prospects, specifically for the development of the optoelectronic devices, providing more possibilities.

## 3 Conclusion

In this paper, a simple Pd/(BA)_2_(MA)_3_Pb_4_Cl_13_/SiO_2_/Si structure of 2D ferroelectric photoelectric memristor was proposed and fabricated. The device exhibits characteristics similar to human synapses, such as PPF, PPD, PTP, LTP, LTD, and “learning to forget” behavior. The device undergoes 10^9^ polarization reversals without significant decay in remaining polarization intensity, and the ferroelectric polarization flip angle is ≈150°. The electrical pulses of the device exhibit varying degrees of current response changes under visible wavelength illumination, allowing the device to simulate an artificial vision system. Interestingly, the device has a high accuracy of 97.15% for handwritten digit recognition by linear conductance values measured by LTP. Meanwhile the optical–electrical reserve pool system can improve 14.55% for the face recognition accuracy. This work demonstrates the potential of (BA)_2_(MA)_3_Pb_4_Cl_13_ materials for use in next‐generation artificial vision chip systems.

## 4 Experimental Section

### Device Fabrication

To prepare the Pd/(BA)_2_(MA)_3_Pb_4_Cl_13_/SiO_2_/Si device, first place a clean *n*‐type Si sheet with 3.4 nm SiO_2_ on the suction cup in the middle of the spin coater. Then, set the rotation speed to 1000 rpm and the rotation time to 30 s, and apply 20 µL solution of 2D (BA)_2_(MA)_3_Pb_4_Cl_13_ perovskite to the SiO_2_/Si surface. Once the spin coating is done, move the substrate to a vacuum drying oven and set the temperature to 60 °C. Allow the substrate to dry for 30 min and repeat the above steps once more. Finally, deposit the Pd top electrode by using the DC magnetron sputtering. That step completes the preparation of Pd/(BA)_2_(MA)_3_Pb_4_Cl_13_/SiO_2_/Si memristor.

### Measurement and Statistics

The 2D (BA)_2_(MA)_3_Pb_4_Cl_13_ perovskite microstructure is characterized by the via X‐ray photoelectron spectroscopy (XPS) and piezoresponse force microscopy (PFM) with a conductive tip (80 N/m, Pt/Ti) was employed. The ferroelectricity of 2D (BA)_2_(MA)_3_Pb_4_Cl_13_ was measured by a ferroelectric tester (TZFE‐200F). All optical and electrical measurements were performed at room temperature using a Keithley 4200 system. Statistical analyses were performed using Origin 2021 software. Other descriptions are provided in the corresponding positions in the main text.

## Conflict of Interest

The authors declare no conflict of interest.

## Supporting information

Supporting Information

## Data Availability

The data that support the findings of this study are available from the corresponding author upon reasonable request.
