# Supplementary material for: Optical–Electrical Coordinately Modulated Memristor Based on 2D Ferroelectric RP Perovskite for Artificial Vision Applications
Source: Adv Sci (Weinh). 2024 Jul 1;11(33):2403150. doi: 10.1002/advs.202403150 (PMC11434019; doi:10.1002/advs.202403150)
Supplement: Supplementary file 1 — Supporting Information [file ADVS-11-2403150-s001.docx]

***Supporting Information***

**Optical-Electrical Coordinately Modulated Memristor Based on 2D Ferroelectric RP Perovskite for Artificial Vision Applications**

Hong Wang^1^, Jialiang Yang^1^, Zheng Yang^1^, Gongjie Liu^1^, Yusong Tang^1^, Yiduo Shao^1^, Xiaobing Yan^1, 2, *^

*^1^ Key Laboratory of Brain-Like Neuromorphic Devices and Systems of Hebei Province, Hebei Key Laboratory of Photo-Electricity Information and Materials, Hebei University, Baoding 071002, China.*

*^2^ Department of Materials Science and Engineering, National University of Singapore, Singapore 117576, Singapore.*

*^*^ Author to whom correspondence should be addressed: yanxiaobing@ime.ac.cn*

| Active Materials | Stimulation | | Synaptic Functions | Image Recognition | |
| --- | --- | --- | --- | --- | --- |
|  | *Optical* | *Electrical* |  | *Database* | *Accuracy* [%] |
| Si NCs/P3HT ^1)^ | √ | — | PPF/PPD/LTP/LTD/SRDP | MNIST | 90.4 |
| *h*-BN/WSe_2_ ^2)^ | √ | — | LTP/LTD | MNIST | >90.0 |
| MoS_2_/*h*-BN ^3^ | √ | √ | LTP/LTD | ECG | 96.1 |
| InSe ^4)^ | √ | √ | PPF/LTP/LTD | MNIST | 65.3 |
| Ge_2_Sb_2_Te_5_ ^5)^ | — | √ | STDP/LTP/LTD | MNIST | 97.8 |
| BTBT-C10/PSBMA ^6)^ | √ | √ | PPF/STP/LTP/STM/LTM | BST | 95.6 |
| MgO/ZnO ^7)^ | √ | √ | LTP/LTD | CIFAR-10 | ~90.0 |
| HfZrO_x_ ^8)^ | — | √ | LTP/LTD | CIFAR-10 | 93.4 |
| Cs_x_FA_y_MA_1-x-y_Pb(I_z_Br_1-z_)_3_ ^9)^ | — | √ | PPF/PPD/LTP/LTD | MNIST | 90.4 |
| InGaAs/HfO_2_ ^10)^ | √ | √ | LTP/LTD | MNIST | 84.0 |
| NiO ^11)^ | √ | — | PPF/PPD/STM/LTM | — | — |
| MoS_2_/ZnO ^12)^ | √ | — | PPF/STM/LTM | — | — |
| BP/CdS ^13)^ | √ | √ | LTP/LTD | MNIST | 94.1 |
| ZnAlSnO/SnO ^14)^ | √ | — | PPF/PPD/STP/LTP/STD/LTD | MNIST | 92.7 |
| Bi_2_O_2_Se/graphene ^15)^ | √ | — | PPF/LTP/LTD/LTM/SRDP | — | — |
| 2D perovskite ^16)^ | √ | — | PPF/STDP | MNIST | 80.0 |
| ReSe_2_ ^17)^ | √ | √ | PTP/LTP/LTD | MNIST | 97.0 |
| (BA)_2_(MA)_3_Pb_4_Br_13_ ^this work)^ | √ | √ | PPF/PPD/LTP/LTD | MNIST/Yale | 97.2/90.9 |

Table S1. Compilation of synaptic devices for image recognition.


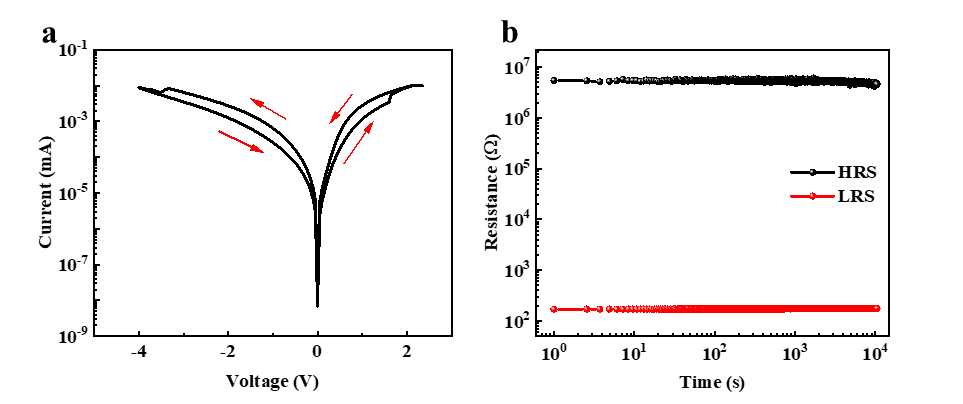


Figure S1. The log diagram *I-V* characteristic curve (a) and high/low resistance retention (b).

**Figure 1**d taking the log to get **Figure S1**a shows that the device has window with good memristor function for the cyclic voltage of 0 V→2 V→0 V→−4 V→0 V applied. The high and low resistance hold-up of the perovskite thin film device was then tested, and the high resistance was about 5.6×10^6^ Ω and the low resistance was about 1.7×10^2^ Ω. The hold-up times were both above 10^4^ s, with good hold-up performance.


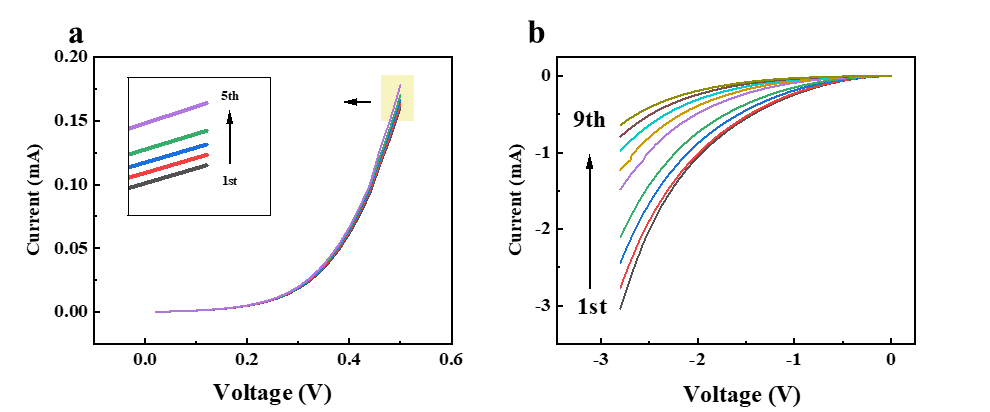


Figure S2. (a) Positive continuous 5-cycle sweeping *I-V* curves. (b) Negative continuous 9-cycle sweeping.

**Figure S2**a is the *I-V* characteristic curve obtained by scanning the device 5 times continuously under the stimulation of positive voltage 0.0 V→0.5 V. From the figure, it can be found that the current value of the *I-V* curve of the latter turn is always higher than that of the former turn under the continuous scanning of positive voltage, which indicates that the current of the device gradually increases and the resistance decreases during the process. In addition, the **Figure S2**b is the *I-V* characteristic curve obtained by applying a negative voltage of 0.0 V→−2.8 V to the device for nine consecutive scans. According to the results, it can be found that the current value of the *I-V* curve of the latter turn is always higher than that of the former turn under the successive scans of negative voltage, meaning that the current of the device gradually decreases and the resistance increases.


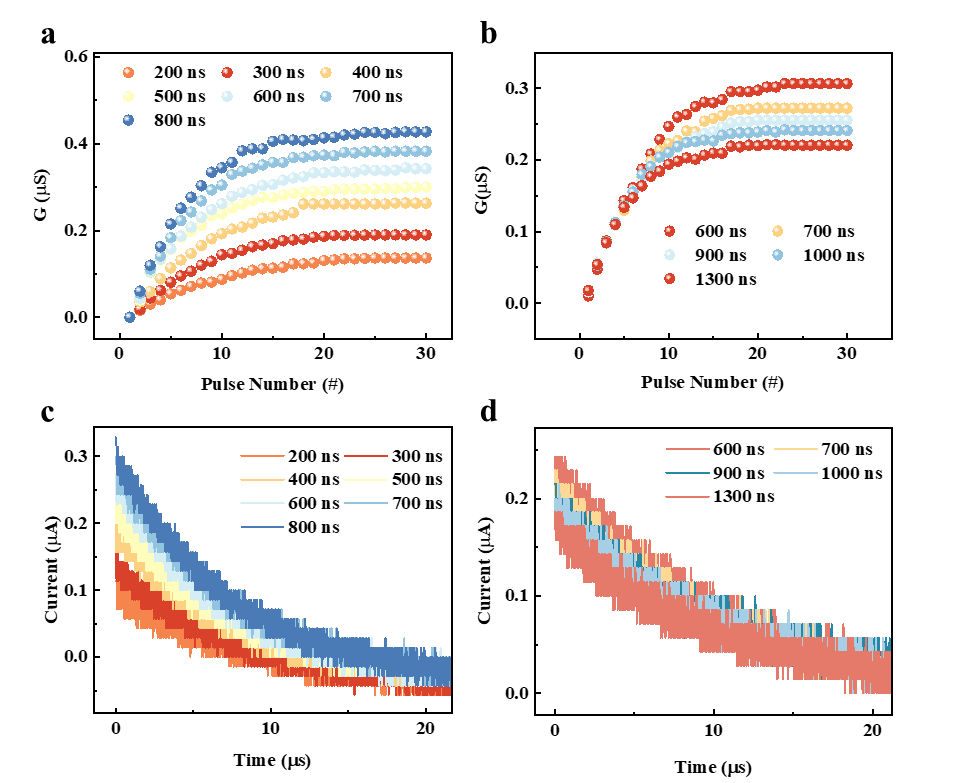


Figure S3. Conductance modulation by pulses of different pulse widths and intervals. (a) Conductance diagram of the device after applying pulses of different pulse widths. (b) Conductance diagram of the device after applying pulses of different intervals. (c) EPSC of the device after applying pulses of different pulse widths. (d) EPSC of the device after applying pulses of different intervals.

**Figure S3** is the application of 30 pulses to the device with a fixed amplitude of 1 V, a fixed interval of 800 ns, and a pulse width varying from 200 ns to 800 ns. The conductance of the device after applying the pulses is shown in **Figure S3**a shows that as the pulse width increases, the device conductance gradually increases, meaning that the device resistance gradually decreases. The corresponding EPSC is shown in **Figure S3**c shows that the EPSC of the device not only becomes larger but also decays more slowly as the pulse width increases. Subsequently, 30 pulses are applied to the device with a fixed amplitude of 1 V, a fixed pulse width of 800 ns, and a pulse interval varying from 600 ns to 1300 ns, as shown in **Figure S3**b, as the pulse interval increases, it can be seen that the device conductivity value gradually decreases, meaning that the device resistance gradually increases. The corresponding EPSC is shown in **Figure S3**d, and the EPSC of the device not only becomes smaller but also decays faster as the pulse interval increases.


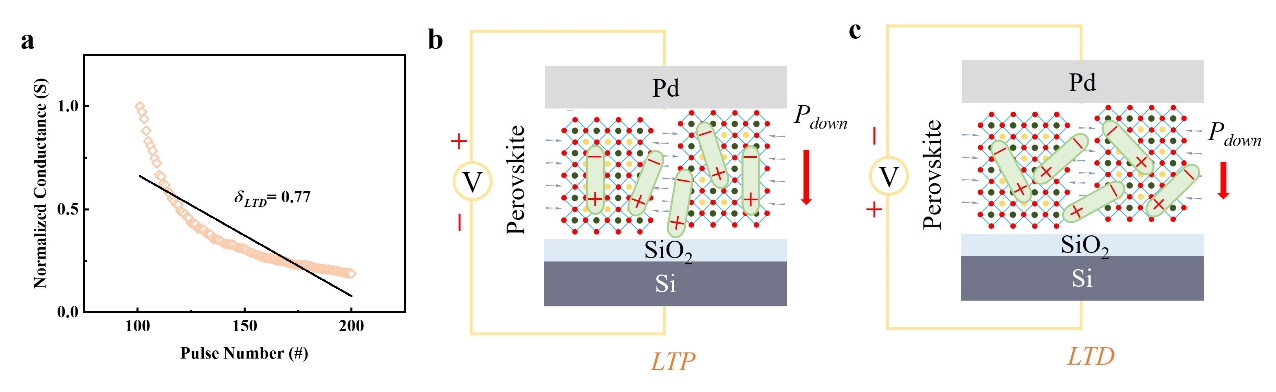


Figure S4. (a) Long-term depression of the (BA)_2_(MA)_3_Pb_4_Br_13_ device. (b, c) The schematic diagram corresponding to the LTP/LTD.


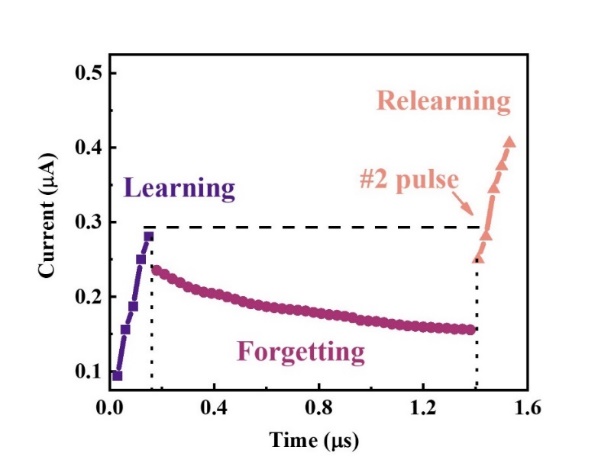


Figure S5. Device learning-forgetting-relearning process.

As **Figure S5** shown first a sequence of 5 positive pulses (pulse amplitude of 3 V, pulse width of 300 ns, and pulse interval of 120 μs) was applied to stimulate the perovskite device for testing, namely the Learning section, it can be seen that the current response increases with the number of pulses and naturally decays if there is no input voltage, as shown in the Forgetting section of the figure. Subsequent application of five more stimulus voltages with the same parameters as the first, as seen in the Relearning section of the figure, shows that this process allows the device to rapidly recover memory with much fewer stimuli than is required for initial learning. The simulates the process of learning to forget, with synaptic weight being significantly enhanced by the first pulse sequence and then spontaneously decaying to intermediate levels in the interval, similar to how information that a person learns that is often partially forgotten after a period of time. To recover the attenuated synaptic weight, only 2 pulses were found to be sufficient during the second stimulus, which is much less than the 5 pulses required to elicit the same synaptic weight enhancement during the first stimulus. These behaviors are similar to the fact that a person usually needs less time to relearn previously remembered lost information and that the relearning process can significantly enhance the stability of memory.


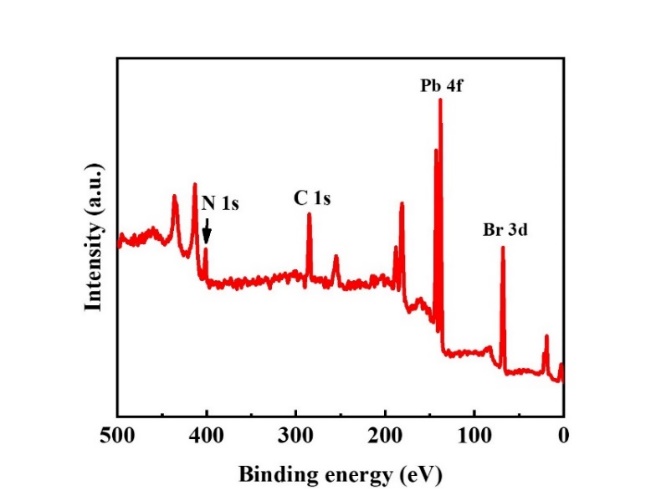


Figure S6. XPS full spectrum.

All spectra were charge-corrected using the C 1s (284.8 eV) peak. **Figure S6** shows the full XPS spectrum of the device with the characteristic peak of N 1s selement appearing at 401.03 eV. The characteristic peaks of Pb 4f and Br 3d and their binding energies are shown in **Figure 2**a-b.


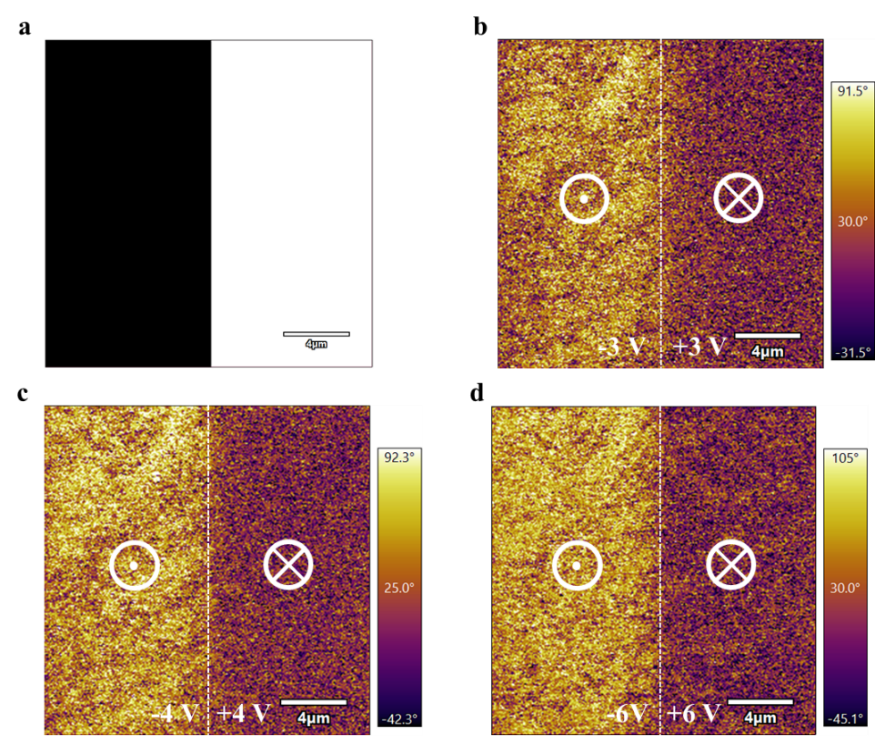


Figure S7. (a) Graphical schematic of PFM applied voltage (+5 V applied in black area and −5 V applied in white area). (b) Phase flip image with PFM applied ±3 V. (c) Phase flip image with PFM applied ±4 V. (d) Phase flip image with PFM applied ±6 V.


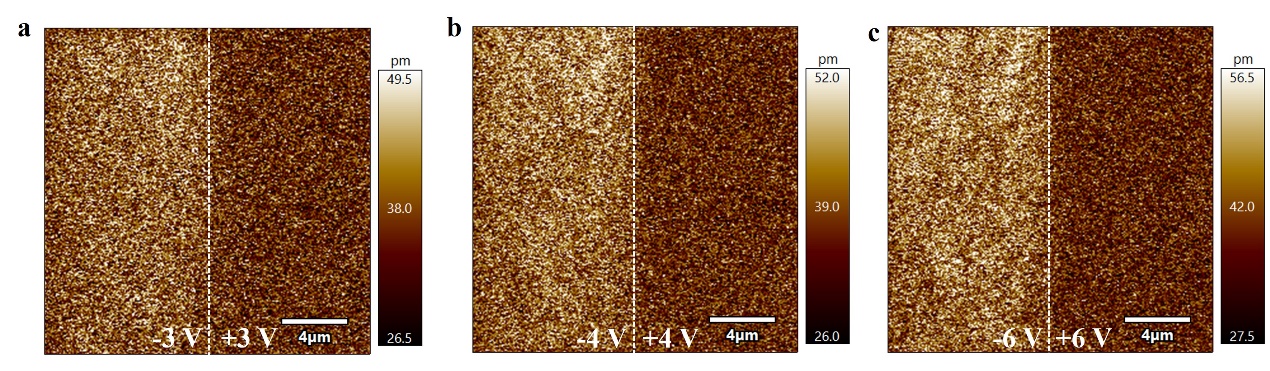


Figure S8. (a) Amplitude image of PFM with ±3 V applied. (b) Amplitude image of PFM with ±4 V applied. (c) Amplitude image of PFM with ±6 V applied.


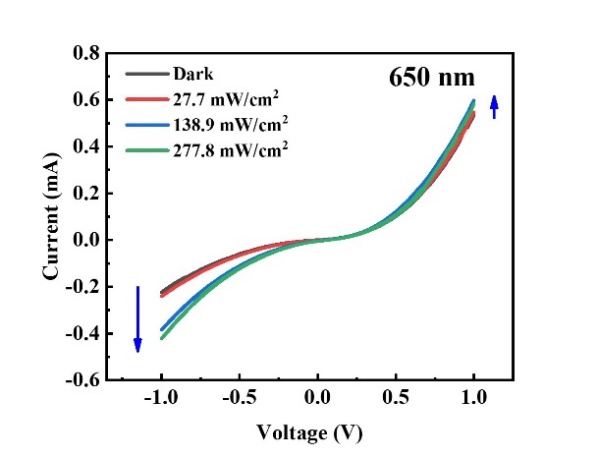


Figure S9. *I-V* curves of the device under the 650 nm light.


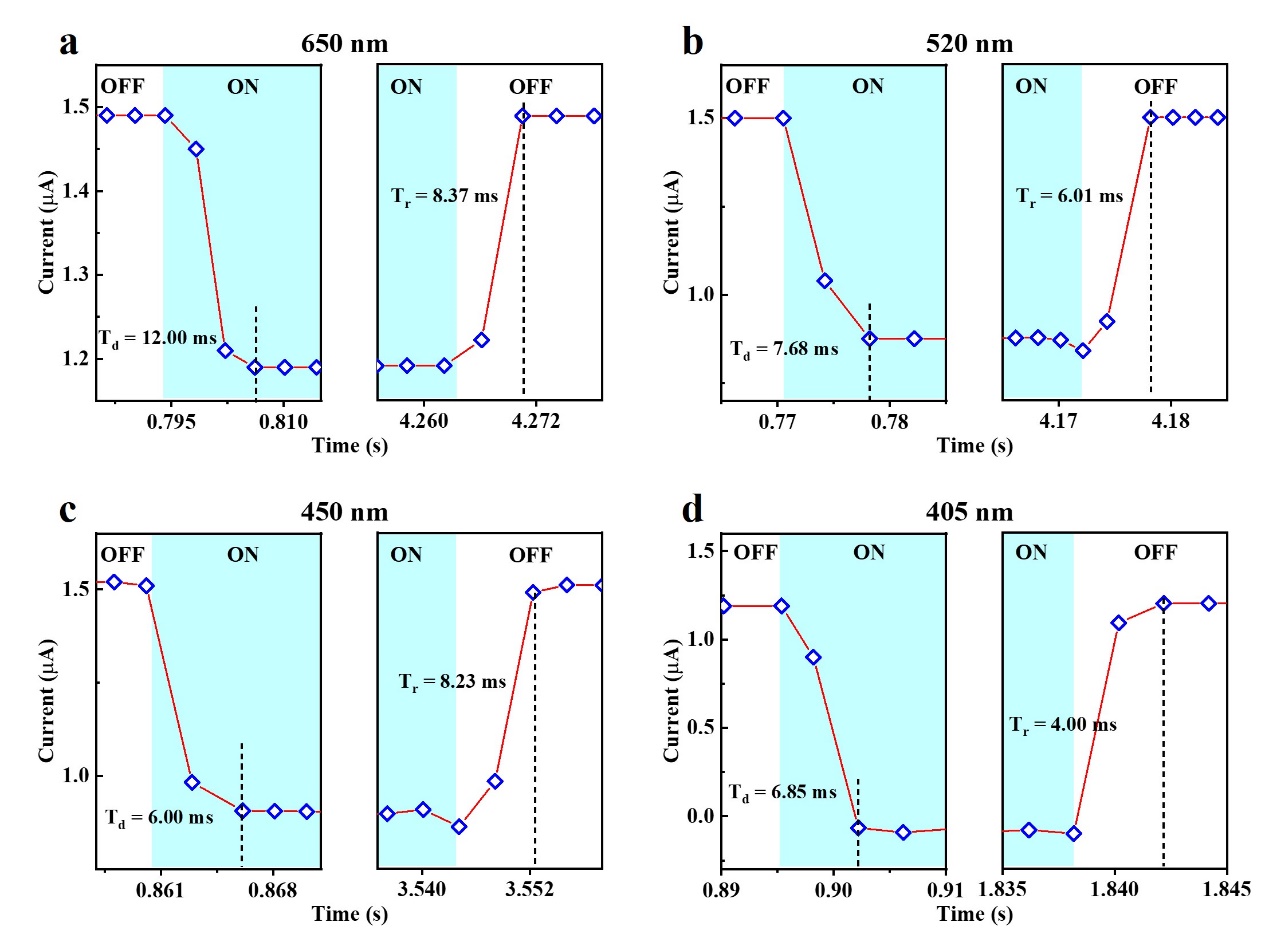


Figure S10. Device optical response speeds under the different wavelength illumination (650 nm, 520 nm, 450 nm and 405 nm).


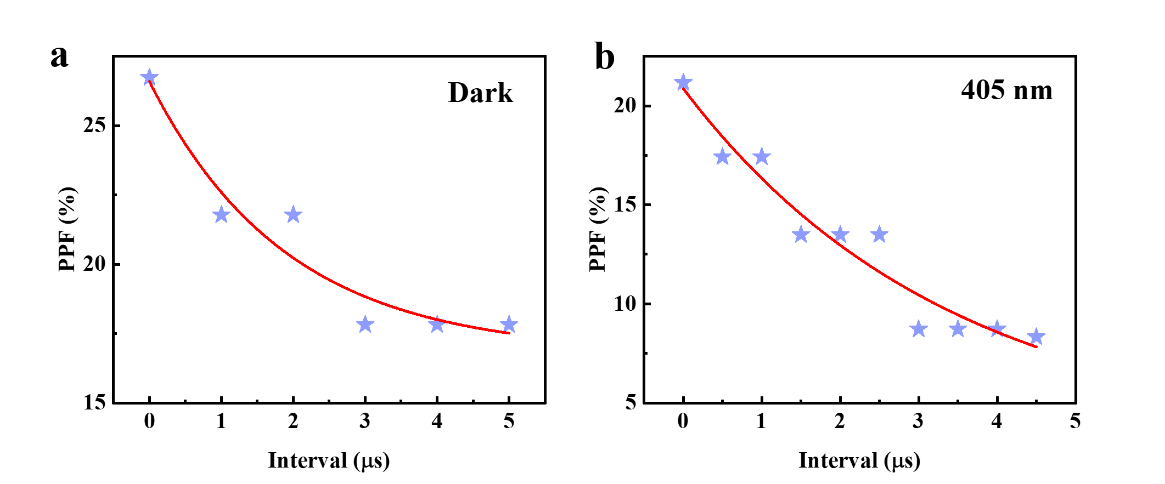


Figure S11. PPF behaviors of the device under the dark and 405 nm wavelength light.

The amplitude of the test pulse is 3 V and the pulse width is 500 ns. As the time interval (*Δt*) between paired pulses decreases, the synaptic weight gradually increases, and the PPF curve fitting equation in the figure is^26^:

G_1_ represents the conductance before the arrival of the first pulse, *G_2_* represents the conductance value after the action of the second pulse, *τ_1_* and *τ_2_* are time fitting constants, and the values of *τ_1_* and *τ_2_* are 1.9 μs and 3.9 μs for the device under dark conditions, and the values of *τ_1_* and *τ_2_* are both ~3.4 μs for the device under 405 nm illumination.


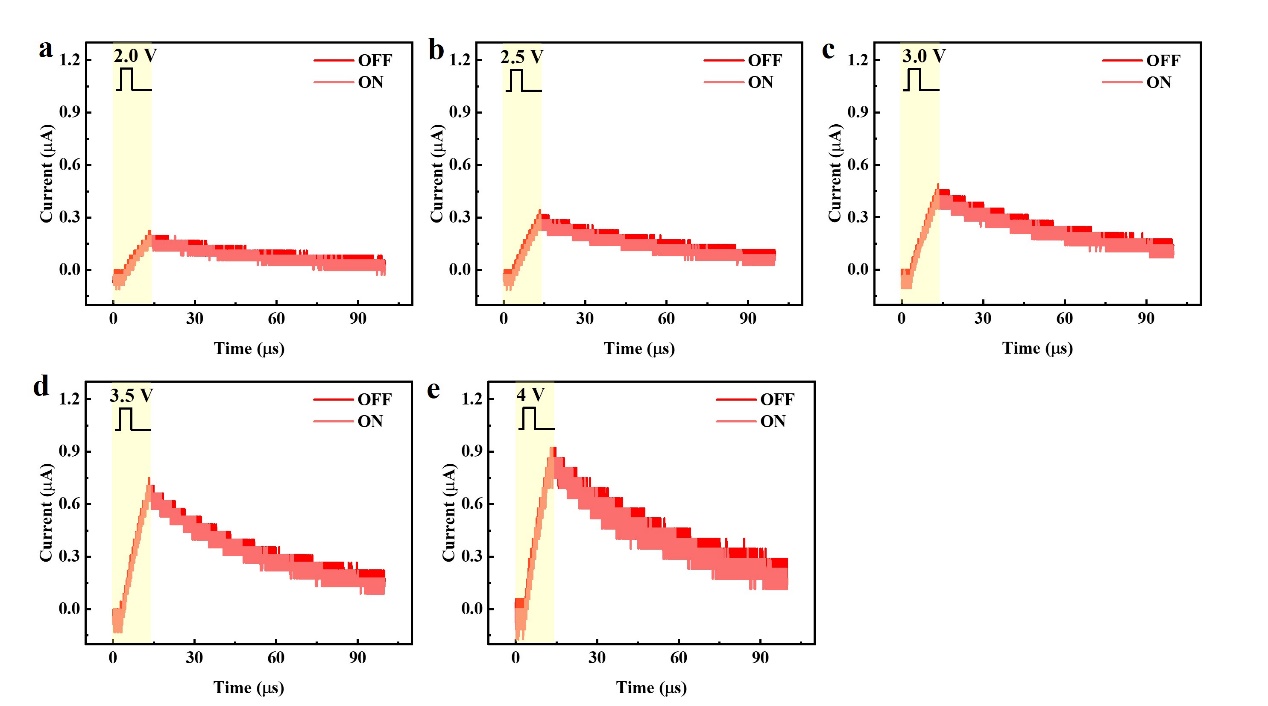


Figure S12. (a-e) Device current response for single pulse at 650 nm and no light (amplitude: 2.0 V, 2.5 V, 3.0 V, 3.5 V, 4.0 V).
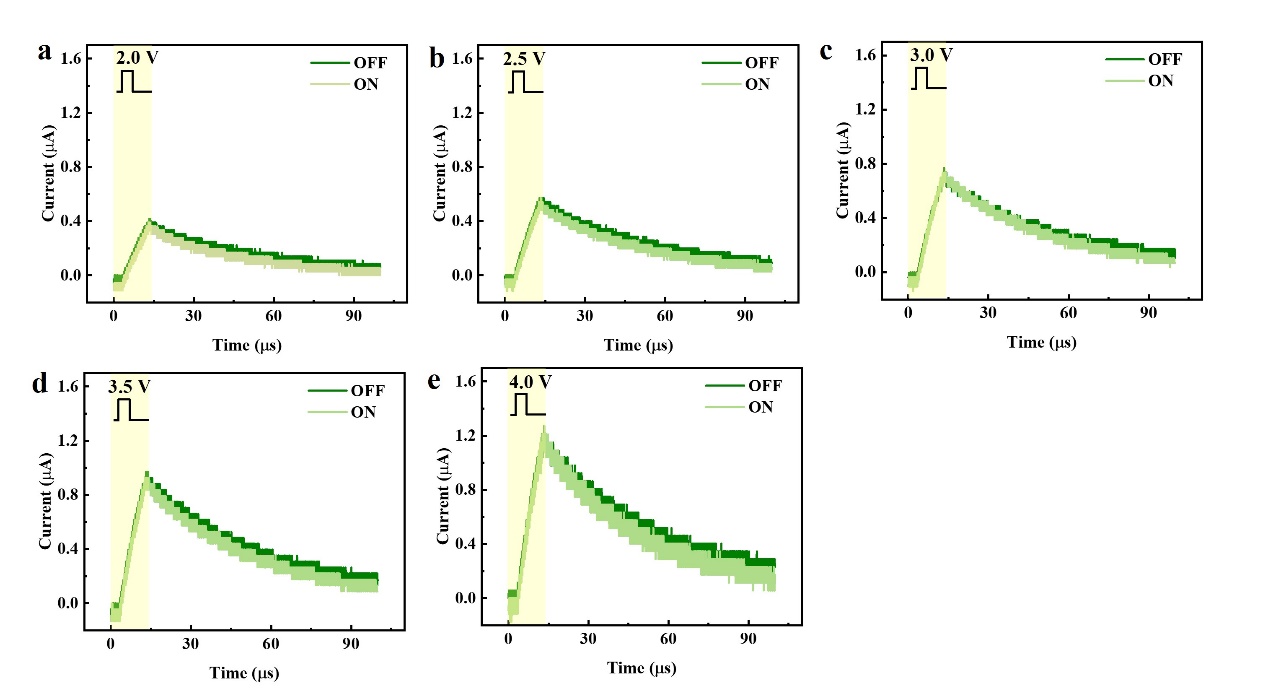


Figure S13. (a-e) Device current response for single pulse at 520 nm and no light (amplitude: 2.0 V, 2.5 V, 3.0 V, 3.5 V, 4.0 V).
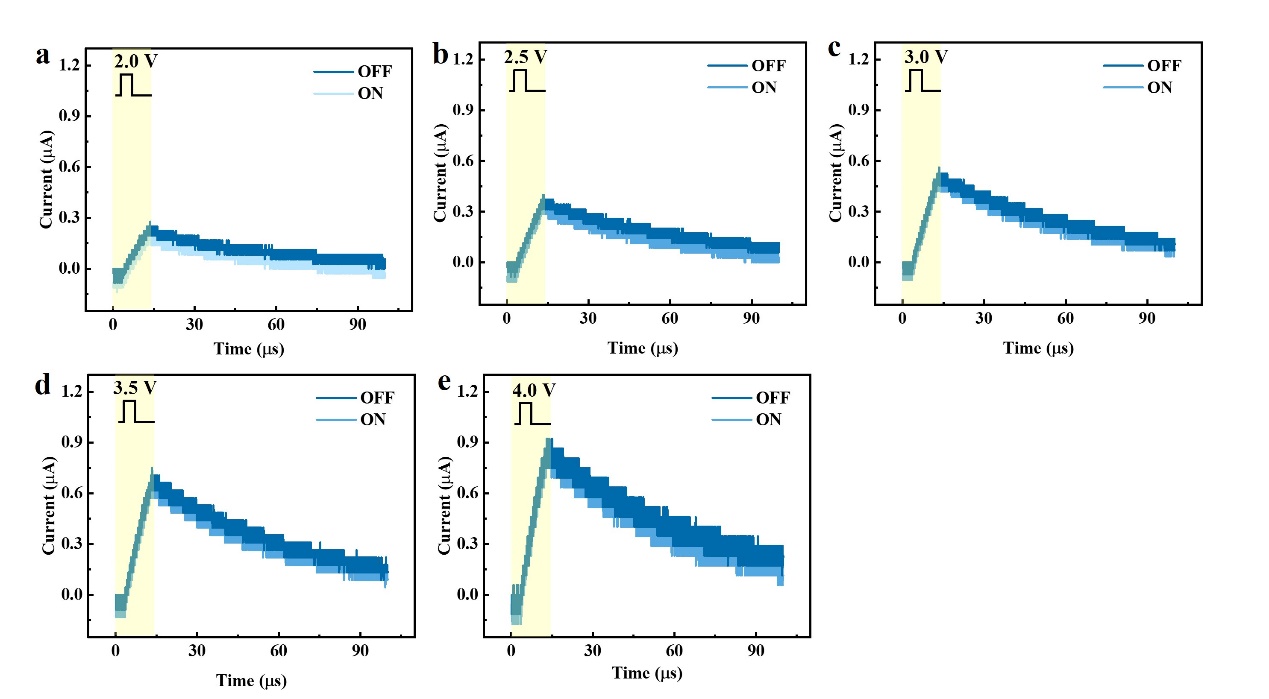


Figure S14. (a-e) Device current response for 405nm and no light case with single pulse (amplitude: 3.0 V, 3.5 V, 4.0 V).

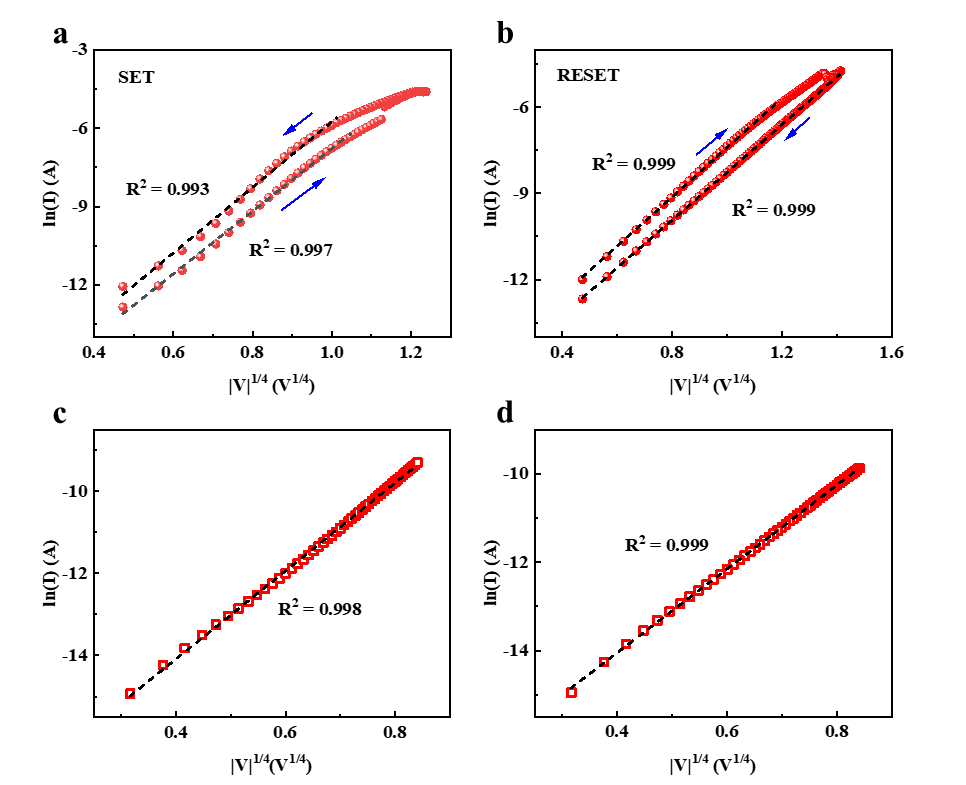


Figure S15. Transmission characteristics of perovskite devices *ln(I)* as a function of *|V|_1/_*_4_ Fig.(a) Device SET process. (b) Device RESET process. (c) Fit of *I-V* positive mechanism under 650 nm illumination. (d) Fit of *I-V* negative mechanism under 650 nm illumination.
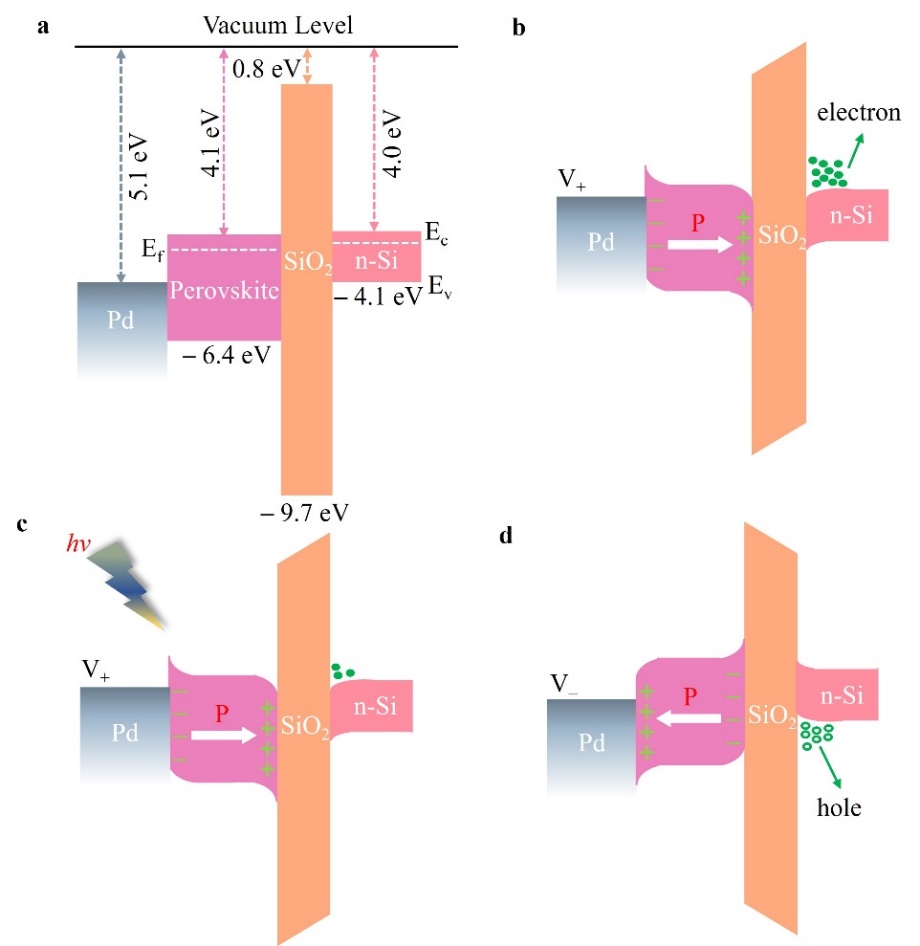


Figure S16. (a) Power function and band gap parameters of Pd/(BA)_2_(MA)_3_Pb_4_Br_13_/SiO_2_/Si structured devices. (b) Polarization state and charge distribution under positive signal. (c) Polarization state and charge distribution under added light. (d) Polarization state and charge distribution under negative signal.

When this memristor is in HRS, when a high enough positive voltage is applied to the top electrode, the perovskite film polarizes downward and electrons in the Pd electrode can be injected into the Pd/perovskite interface and trapped into the trap. This makes the Schottky barrier height at the interface low. When the trap is filled with enough electrons, the Schottky barrier becomes negligible and more electrons can be easily injected, allowing the device to reach the LRS, as shown in **Figure S16**b. The energy band diagram of the perovskite device after adding light is shown in **Figure S16**c. At the LRS, when a negative voltage is applied to the top electrode Pd, the release of electrons occurs and the perovskite film polarizes upward, which causes the height of the Schottky barrier between the Pd/perovskite film interface to become higher and the device enters the HRS, as shown in **Figure S16**d.


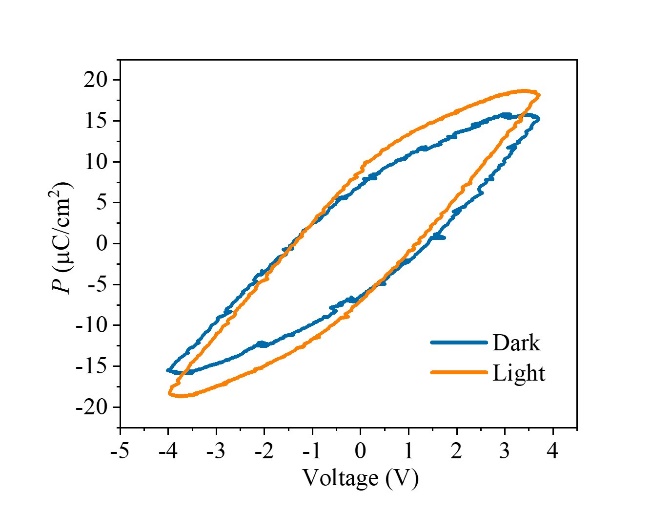


**Figure S17** Polarization-electric-field (*P-E*) loops of 2D RP (BA)_2_(MA)_3_Pb_4_Br_13_ perovskite under the dark and illumination.


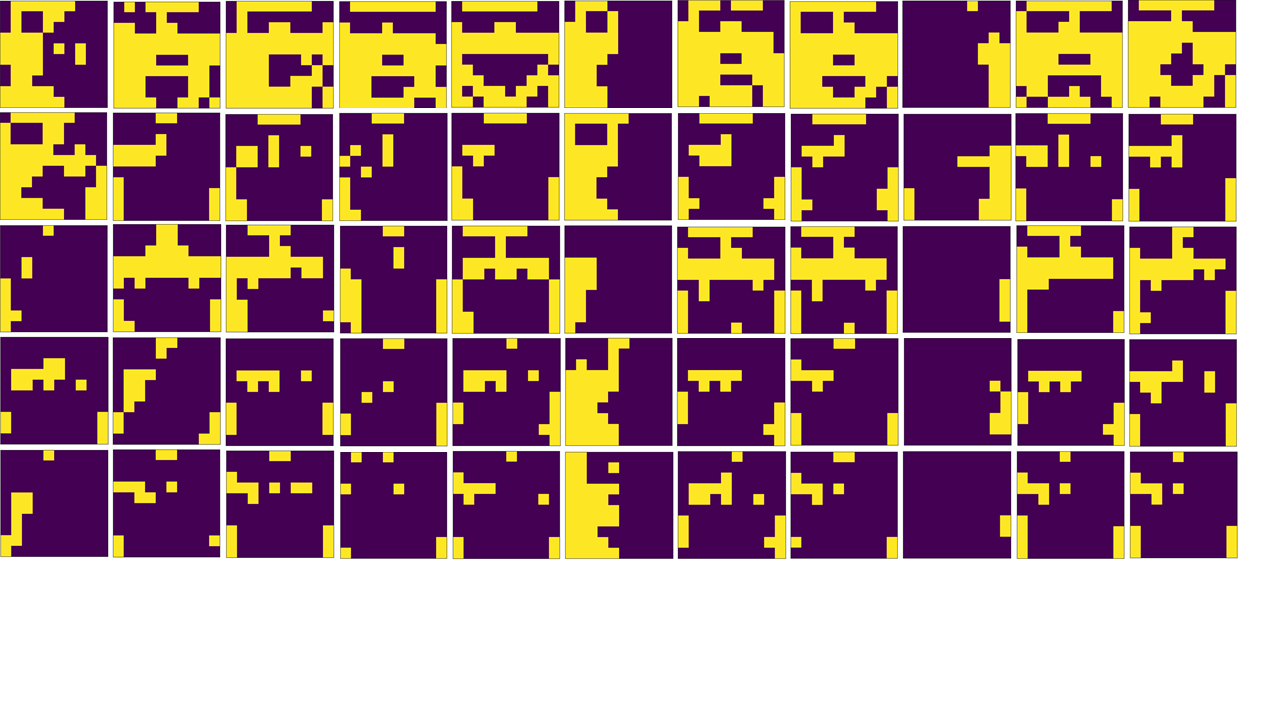


**Figure S18** Results of the image scaling process on the faces of five individuals.

**Reference**

[1] Y. Wang, Y. Zhu, Y. Li, Y. Zhang, D. Yang, X. Pi, *Adv. Funct. Mater.* **2022**, *32*(1), 2107973.

[2] S. Seo, S. H. Jo, S. Kim, J. Shim, S. Oh, J. H. Kim, *et al. Nat. Commun.* **2018**, *9*(1), 5106.

[3] S. Oh, J. J. Lee, S. Seo, G. Yoo, J. H. Park, *NPJ 2D Mater. Appl.* **2021**, *5*(1), 95.

[4] F. S. Yang, M. Li, M. P. Lee, I. Y. Ho, J. Y. Chen, H. Ling, *et al.* *Nat. Commun.* **2020**, *11*(1), 2972.

[5] I. Boybat, M. Le Gallo, S. R. Nandakumar, T. Moraitis, T. Parnell, T. Tuma, *et al.* *Nat. Commun.* **2018**, *9*(1), 2514.

[6] X. Wu, S. Shi, B. Liang, Y. Dong, R. Yang, R. Ji, *et al. Sci. Adv.* **2024**, *10*(16), eadn4524.

[7] B. Dang, L. Ma, L. Yan, S. Wang, K. Liu, L. Xu, *et al.* *IEEE Electr. Device Lett.* **2020**, *41*(11), 1641.

[8] Y. Zhu, Y. He, C. Chen, L. Zhu, H. Mao, Y. Zhu, *et al.* *Appl. Phys. Lett.* **2022**, *120*(11), 113504*.*

[9] B. Cai, Y. Huang, L. Tang, T. Wang, C. Wang, Q. Sun, *et al. Adv. Funct. Mater.* **2023**, *33*(46), 2306272.

[10] B. Bae, M. Park, D. Lee, I. Sim, K. Lee, *Adv. Optic. Mater.* **2023**, *11*(3), 2201905.

[11] C. Lu, J. Meng, J. Song, T. Wang, H. Zhu, Q. Q. Sun, *et al.* *Nano Lett.* **2024**, *24*, 1667.

[12] J. Zheng, Y. Du, Y. Dong, X. Shan, Y. Tao, Y. Lin, *et al. Appl. Phys. Lett.***2024**, *124*(13).

[13] C. Zhu, H. Liu, W. Wang, *et al.* *Light: Sci. Appl.* **2022**, *11*(1), 337.

[14] R. Yang, Y. Wang, S. Li, D. Hu, Q. Chen, F. Zhuge, *et al. Adv. Funct. Mater.* **2024**, *34*(10), 2312444.

[15] C. M. Yang, T. C. Chen, D. Verma, L. J. Li, B. Liu, W. H. Chang, C. S. Lai, *Adv. Funct. Mater.* **2020**, *30*(30), 2001598.

[16] H. Tian, X. Wang, F. Wu, Y. Yang, T. L. Ren, *In* *2018 IEEE International Electron Devices Meeting (IEDM)*. IEEE, **2018**, 38.

[17] H. Wang, J. Yang, Z. Wang, Y. Shao, Y. Tang, J. Guo, X. Yan, *Appl. Phys. Rev.* **2024**, *11*(1).
